# Technology for Rapid Detection of Cyromazine Residues in Fruits and Vegetables: Molecularly Imprinted Electrochemical Sensors

**DOI:** 10.3390/bios12060414

**Published:** 2022-06-14

**Authors:** Sihua Peng, Aqiang Wang, Yuyang Lian, Jingjing Jia, Xuncong Ji, Heming Yang, Jinlei Li, Shuyan Yang, Jianjun Liao, Shihao Zhou

**Affiliations:** 1 Key Laboratory of Germplasm Resources Biology of Tropical Special Ornamental Plants of Hainan Province, College of Forestry, Hainan University, Haikou 570228, China; pengsihua@126.com (S.P.); fly88200939@163.com (A.W.); l18876114522@126.com (Y.L.); yangheming97@163.com (H.Y.); jlli1013@163.com (J.L.); ysy000567@foxmail.com (S.Y.); 2 College of Plant Protection, Hainan University, Haikou 570228, China; 3 Institute of Plant Protection, Hainan Academy of Agricultural Sciences (Research Center of Quality Safety and Standards for Agricultural Products of Hainan Academy of Agricultural Sciences), Haikou 571199, China; j9405136318@163.com (J.J.); insects99@163.com (X.J.)

**Keywords:** edible agricultural products, rapid detection of pesticide residues, molecular imprinting sensor, cyromazine

## Abstract

Cyromazine is an insect growth regulator insecticide with high selectivity and is widely used in the production and cultivation of fruits and vegetables. In recent years, incidents of excessive cyromazine residues in food have occurred frequently, and it is urgent to establish an accurate, fast, and convenient method for the detection of cyromazine residues to ensure the safety of edible agricultural products. To achieve rapid detection of cyromazine residues, we prepared a molecularly imprinted electrochemical sensor for the detection of cyromazine residues in agricultural products. Samples of tomato (*Lycopersicon esculentum* Miller), cowpea (*Vigna unguiculata*), and water were tested for the recovery rate of cyromazine. The results showed that the concentration of cyromazine showed a good linear relationship with the peak response current of the sensor developed in this study. The lower limit of detection for cyromazine was 0.5 µmol/L, and the sensor also had good reproducibility and interference resistance. This paper can be used as a basis for the study of methods for the detection of cyromazine residues in edible agricultural products.

## 1. Introduction

Cyromazine is an insect growth regulator insecticide with strong systemic, stomach poisoning, and contact killing abilities. It is currently widely used in the control of fly pests in fruits and vegetables such as cowpea (*Vigna unguiculata*) and tomato (*Lycopersicon esculentum* Miller) [1,2]. Cyromazine itself has low toxicity to humans, but its degradation product melamine, when ingested in large quantities, can cause irreversible damage to the kidneys and is a serious health hazard [3]. As modern technology and analytical methods continue to improve, there is a need for greater accuracy and sensitivity in the detection of cyromazine in food [4]. To prevent cyromazine residues in food from causing harm to consumers, it is necessary to develop a simple and rapid detection method for cyromazine residues with high sensitivity and accuracy.

At present, the relatively mature technologies for the detection of cyromazine residues in food include liquid chromatography–tandem mass spectrometry (LC-MS), ultra-high-performance liquid chromatography (UPLC), enzyme-linked immunosorbent assay (ELISA), and high-performance liquid chromatography (HPLC) [5,6,7]. Although the above-mentioned methods have high accuracy and sensitivity, the high equipment cost and complicated operation hinder their further development. Therefore, there is an urgent need to develop an analytical method for pesticide residue determination with good sensitivity and selectivity, low cost, easy portability, and large-scale use.

Molecularly imprinted polymers (MIPs) are an advanced means of overcoming the limitations of traditional detection methods described above. MIPs are artificial recognition materials that are complementary in size and shape to template molecules and can be used for the specific target purpose of molecule identification [8,9]. As a novel material and bionic molecular recognition element, MIPs have unique advantages over traditional enzymes, antibodies, and receptors, such as excellent stability, high selectivity, ease of preparation, stability in organic solvents, and broad compatibility with many scientific fields [10,11,12]. In addition to their excellent identification properties, MIPs offer the advantages of low cost, heat and pressure resistance, storage stability, and suitability in harsh chemical media. Compared to conventional detection techniques, electrochemical detection methods are simple, low cost, and have a fast response time, thus offering significant advantages for pesticide residue detection in food [13,14,15]. Pan et al. used coumarin as a template molecule and MIP as a recognition element to prepare a chemiluminescence sensor on a 96-well microplate for the determination of organophosphorus residues in milk samples. The synthesized MIP can specifically recognize seven organic phosphorus [16]. The lowest detection limit of the sensor was 1 pg/mL, and the recoveries for seven organophosphorus species ranged from 86.1% to 86.5%. This shows that the detection of pesticide residues in food by molecularly imprinted polymers has broad application prospects [17,18]. There is no report on the use of molecularly imprinted sensors for the detection of cyromazine residues in food. In this study, electrochemical detection of cyromazine was achieved for the first time, which provides a reference for the rapid detection of triazine pesticides and other pesticides in food. In this study, a portable molecular imprinting sensor for cyromazine was prepared by electrochemistry combined with a molecular imprinting technique, using a gold nanoparticle-modified electrode, cyromazine as a template molecule, α-methacrylic acid as a functional monomer, trimethylolpropane trimethacrylate as a crosslinking agent, and HCl as an eluent under specific conditions, and the method was applied to the rapid analysis and detection of cyromazine in tomato, cowpea, and water samples.

## 2. Materials and Methods

### 2.1. Materials and Reagents

Cyromazine was purchased from Zhengzhou Labor Agrochemicals Co., Ltd., Zhengzhou, China); concentrated hydrochloric acid, sodium dihydrogen phosphate, dibasic sodium phosphate, glutaric dialdehyde, and potassium chloride were purchased from Sinopharm Chemical Reagent Co., Ltd. (Shanghai, China); potassium hexacyanoferrate(II) was purchased from Xilong Scientific Co., Ltd. (Guangzhou, China); potassium ferricyanide was purchased from Guangzhou Chemical Reagent Factory (Guangzhou, China); α-methacrylic acid was purchased from Meryer (Shanghai, China) Chemical Technology Co., Ltd. (Shanghai, China); trimethylolpropane trimethacrylate was purchased from Hubei Shishun Bio-Technology Co., Ltd. (Huanggang, China); acetonitrile was purchased from Shanghai Macklin Biochemical Technology Co., Ltd. (Shanghai, China). All the above reagents were analytically pure. Cowpea and tomato were purchased at a nearby farmers’ market. Chloroauric acid (99%) was purchased from Hubei Guangao Biotechnology Co., Ltd. (Wuhan, China). Water samples were collected from paddy fields (19.507036° N, 109.504222° E), rivers (19.513888° N, 109.4917.2° E), and botanical gardens (19.5124° N, 109.4988° E).

### 2.2. Instrumentations and Equipment

Electrochemical workstation (CHI660E, CH Instruments Ins., Austin, TX, USA), screen-printed electrodes (Qingdao Botan Technology Co., Ltd., Qingdao, China), 1/10,000 analytical balance (Quintix124-1CN, Sartorius, Germany), Neofuge 23R high-speed refrigerated centrifuge (Likang Biomedical Technology Holdings Co., Ltd., Hong Kong, China), FST-111-TH100 ultra-pure water machine (Thermo, Waltham, MA, USA).

### 2.3. Preparation of Solutions and Samples

#### 2.3.1. Preparation of Pesticide Standard Solutions

First, 1.2374 g of cyromazine was placed in a 1000 mL volumetric flask, and a 6 µmol/L pesticide standard solution with phosphate-buffered saline (PBS) (pH = 7.4) solvent was prepared and stored in a room-temperature environment. The 6 µmol/L pesticide stock solutions were then diluted with PBS into a series of pesticide standard solutions of 0.5, 1.0, 1.5, 2.0, 2.5, and 3.0 µmol/L, respectively.

#### 2.3.2. Pretreatment of Test Samples

Tomato samples: Prepared tomatoes were washed and dried, chopped, and beaten well. Thirty grams of the homogenate was weighed and added to 30 mL of acetonitrile solution and vortexed for 3 min, then poured into a 50 mL centrifuge tube and centrifuged at 8000 r/min for 5 min, and the supernatant was filtered for use. The corresponding amount of cyromazine was added to the supernatant to prepare sample solutions containing 1, 2, and 3 μmol/L cyromazine. Cowpea samples were treated the same as tomatoes.

Water samples: The sampling bottles were first rinsed with water and filled carefully so that they overflowed to avoid trapping air bubbles in the sealed bottle. The samples were shipped in a refrigerated box with an ice pack. Container preparation included washing with detergent, rinsing with tap and ultra-pure water, and finally air drying. After the samples were transported to the laboratory, they were stored at 4 °C, and the collected water samples were simply filtered within 48 h, and then the corresponding amount of cyromazine was added to prepare sample solutions containing 1, 2, and 3 μmol/L cyromazine.

### 2.4. Preparation of the Modifier

HAuCl_4_ stock solution: 0.5 mol/L H_2_SO_4_ was used as a solvent and a quantitative amount of HAuCl_4_ was added to configure a deposition solution containing 0.2% HAuCl_4_.

Cyromazine and α-methacrylic acid stock solution: Cyromazine and α-methacrylic acid stock solution was prepared with PBS solvent in a molar ratio of 1:4. Then, 0.4 µmol α-methacrylic acid was added to a 100 mL volumetric flask and mixed with ultrasound for 30 min, 0.1 µmol of cyromazine stock solution was added, and then PBS solution was added to increase the volume to 100 mL, sonicated for 1 h, and stored at a temperature of 2 °C.

### 2.5. Preparation of Molecularly Imprinted Sensors

**Figure 1:**
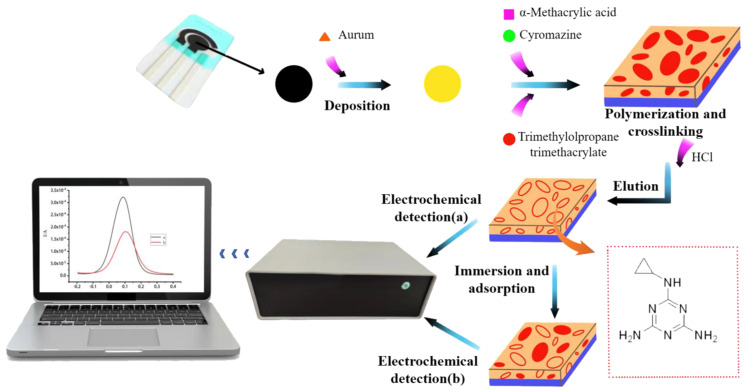
Flow chart of sensor development and testing. (**a**) The prepared molecularly imprinted sensor. (**b**) Molecularly imprinted sensor soaked in the sample solution.

Referring to the methods of preparing electrodes by Shi et al. and Li et al. [19,20] and optimizing them, 80 µL of 0.2% HAuCl_4_ was dropped on the working surface of the electrode, and deposited by the potentiostat method (voltage: −0.25 V) for 3 min. The surface was rinsed with water and dried. Then, 80 µL of the polymerization solution of cyromazine and α-methacrylic acid was added to the surface of the dried electrode and deposited at a voltage of −1.0 V for 5 min. At this time, the surface of the electrode was simultaneously polymerized with cyromazine and α-methacrylic acid, and the electrode was dried. After that, 10 µL of 2 mg/L trimethylolpropane trimethacrylate solution was added to crosslink cyromazine and α-methacrylic acid on the electrode surface. After drying for 12 h, 80 µL of 1% HCL solution was added to elute for 15 min under the condition of a potential range of −0.4 to +0.8 V to obtain a molecularly imprinted sensor. The sensor prepared with the polymer solution without cyromazine was used as the non-molecularly imprinted sensor (NIP). Figure 1 is a flow chart of the development and detection of the sensor.

### 2.6. Electrochemical Characterization and Performance Testing of Sensors

#### 2.6.1. CV and EIS Characterization of Sensors

The homemade sensor was immersed in a 0.1 mol/L KCl solution containing 5.0 mmol/L [K_3_Fe(CN_6_)] and subjected to cyclic voltammetry (CV) scan for 2 segs at a potential range of −0.4 to +0.6 V to obtain the cyclic voltammogram of the sensor; the electrochemical impedance spectrum (EIS) of the sensor was obtained using the AC impedance method between 10^−1^ and 10^−5^ Hz.

#### 2.6.2. Scanning Electron Microscope Characterization of Sensors

The homemade sensor was scanned under a scanning electron microscope to obtain a scanning electron microscope image of the sensor, and its surface was observed and analyzed.

#### 2.6.3. DPV Performance Testing of Sensors

The prepared sensor was tested using differential pulse voltammetry (DPV) using 5.0 mmol/L [K_3_Fe(CN_6_)] and 0.1 mol/L KCL solution as the electrolyte solution to record the peak current (*I*_0_) at this time [21]. Subsequently, the electrodes were immersed in a solution containing different concentrations of cyromazine from high to low, soaked for 20 min, and then taken out to dry, and the electrode peak current was tested with differential pulse voltammetry (DPV) at this time, which is recorded as *I*. The relative suppression (*I*%) of different concentrations of cyromazine on the sensor was calculated using Equation (1):

(1) $$I\%=\frac{I_{0}-I}{I_{0}}\times 100\%.$$

Note: *I*_0_: differential pulse voltammetry peak current of the sensor without pesticide immersion; *I*: differential pulse voltammetry peak current of the sensor with different concentrations of pesticide immersion; *I*%: relative suppression of this sensor with different concentrations of cyromazine.

#### 2.6.4. Repeatability Testing

The prepared sensor was soaked in PBS buffer solution containing 2 μmol/L of cyromazine, taken out and dried after 20 min, and 60 μL of 0.1 mol/L KCL solution containing 5.0 mmol/L [K_3_Fe(CN_6_)] was added to the working area of the sensor, and the differential pulse voltammetry (DPV) method was used to record the peak current value. The above operation was repeated six times and the relative standard deviation (RSD) was calculated.

#### 2.6.5. Interference Test

Two pesticides, atrazine and metolachlor, which have a similar structure to cyromazine, were selected as the pesticides for resistance to interference. To 1 μmol/L of cyromazine solution, atrazine and metolachlor were added to form a mixture of 5 μmol/L, 10 μmol/L, 20 μmol/L, and 30 μmol/L of cyromazine solution, respectively, and this mixture was used as the interference solution. The sensor was immersed in the above solutions in a gradient order for 20 min, removed, and dried, then 60 μL of 0.1 mol/L KCl solution containing 5.0 mmol/L [K_3_Fe(CN_6_)] was added dropwise and scanned by differential pulse voltammetry, and the peak current values were recorded.

#### 2.6.6. Actual Sample Recovery Testing

The sensor was immersed in the sample solution to be tested at different concentrations of cyromazine, left to adsorb for 20 min, and then removed and dried, then 60 μL of 0.1 mol/L KCl solution containing 5.0 mmol/L [K_3_Fe(CN_6_)] was dropped and scanned by differential pulse voltammetry, the peak current values were recorded, three replicates were made for each sample at each concentration, and the recoveries and relative standard deviations were calculated.

## 3. Results and Analysis

### 3.1. Analysis of the Results of Electrochemical Characterization

**Figure 2:**
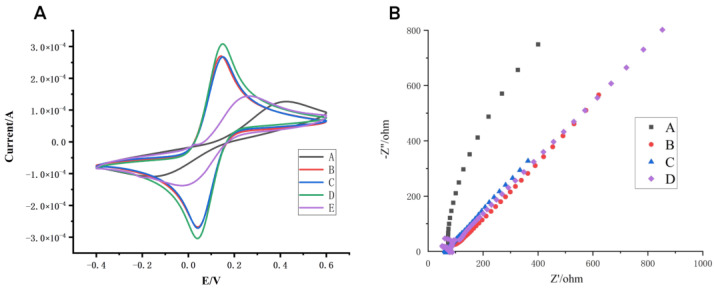
Structural characterization of the sensor in 5.0 mmol/L [K_3_Fe(CN_6_)] solution in 0.1 mol/L KCl. (**A**) Cyclic voltammogram (CV): A bare electrode CV (bare SPCE), B electrode after deposition of HAuCl_4_ solution, C electrode after polymerizing cyromazine molecular polymer, D electrode after elution, E electrode (CK) after elution of non-imprinted sensor. (**B**) Electrochemical impedance spectroscopy (EIS): A bare electrode, B electrode after deposition of HAuCl_4_ solution, C electrode after polymerizing cyromazine molecular polymer, D electrode after elution.

Figure 2A shows the result of the cyclic voltammetry in the sensor preparation steps where, after the screen-printed electrode is modified with chloroauric acid, the peak current of the electrode increases significantly, indicating that the gold nanoparticles are successfully deposited on the surface of the electrode, thereby increasing the conductivity of the electrode. When the polymer of cyromazine and α-methacrylic acid is deposited on the surface of the electrode, the presence of the polymer hinders the diffusional permeability of the redox marker, resulting in a decrease in the peak current of the electrode. After being eluted by HCL, some of the cyromazine molecules in the polymer were eluted, the gap between the polymer molecules became larger, and the electron transport was accelerated. Non-molecularly imprinted electrochemical sensors cannot crosslink to form long chains due to the absence of the participation of cyromazine in the preparation process, resulting in the inability of the polymerization solution to crosslink to form a long chain, which makes the gap between molecules smaller, resulting in a weakening of the conductivity of the sensor. After HCL elution, the peak current of the molecularly imprinted electrochemical sensor is greater than that of the non-molecularly imprinted electrochemical sensors, indicating that the blotting site on the surface of the sensor has good recognition performance for cyromazine.

The electrochemical impedance spectroscopy was performed on the above electrodes respectively, and the results are shown in Figure 2B. The results showed that the electron transport was promoted and the impedance spectrum radius was significantly reduced after chloroauric acid deposition. After the polymer was deposited and eluted, the impedance spectrum radius increased, indicating that the cyromazine and the α-methacrylic acid polymer were successfully bound to the sensor.

### 3.2. Characterization Results of the Sensors Using Scanning Electron Microscopy

**Figure 3:**
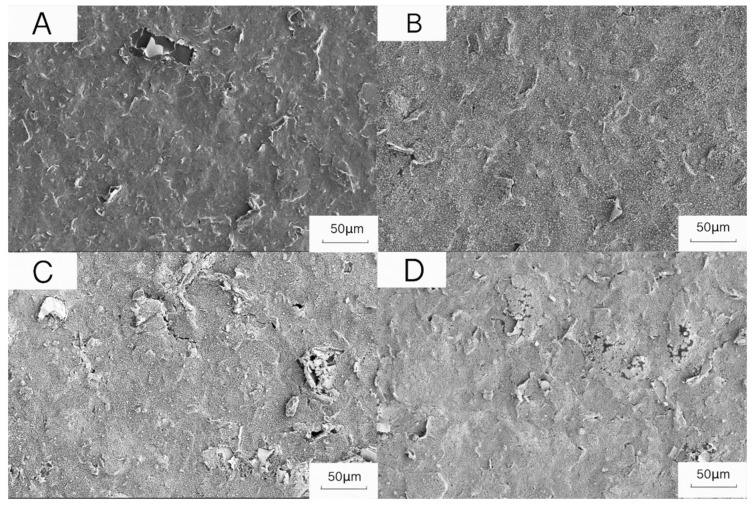
Scanning electron micrograph of the sensor. (**A**) Bare electrode CV (bare SPCE), (**B**) electrode after deposition of HAuCl_4_ solution, (**C**) electrode after polymerizing cyromazine molecular polymer, (**D**) electrode after elution.

The surface of the electrode modified with chloroauric acid had many small particles (Figure 3B) and became smooth, indicating that the gold particles were successfully assembled on the sensor. After elution with 1% HCl, the electrode surface became flat and smooth, and the surface lumps were reduced (Figure 3D), indicating that the cyromazine molecules were successfully eluted.

### 3.3. Performance Test Results of Sensors

**Figure 4:**
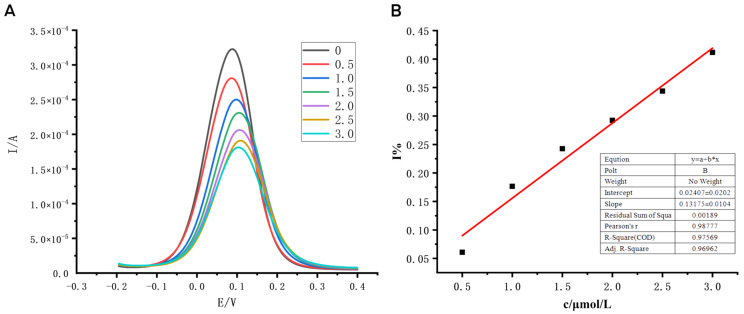
Standard curve and relative suppression curve of cyromazine solution with different concentrations. Note: (**A**) DPV curve of the molecularly imprinted sensor with different concentrations of cyromazine, the (**B**) curve of relative suppression of the molecularly imprinted sensor with different concentrations of cyromazine; 0, 0.5, 1.0, 1.5, 2.0, 2.5, 3.0 are different concentrations of cyromazine, where the concentration unit is μmol/L.

The prepared sensor was used to test solutions containing different concentrations of cyromazine, the results are shown in Figure 4A, and the relative suppression curve of the cyromazine solution was drawn in combination with the peak current of the sensor (Figure 4B). It can be seen that the peak current of the sensor is negatively correlated with the concentration of cyromazine, and the specific performance is that with the increase in the concentration of cyromazine, the corresponding peak current decreases. Therefore, in a certain concentration range, there is a good linear relationship between the response current of the sensor and the pesticide concentration.

### 3.4. Results of Repeatability Tests

**Figure 5:**
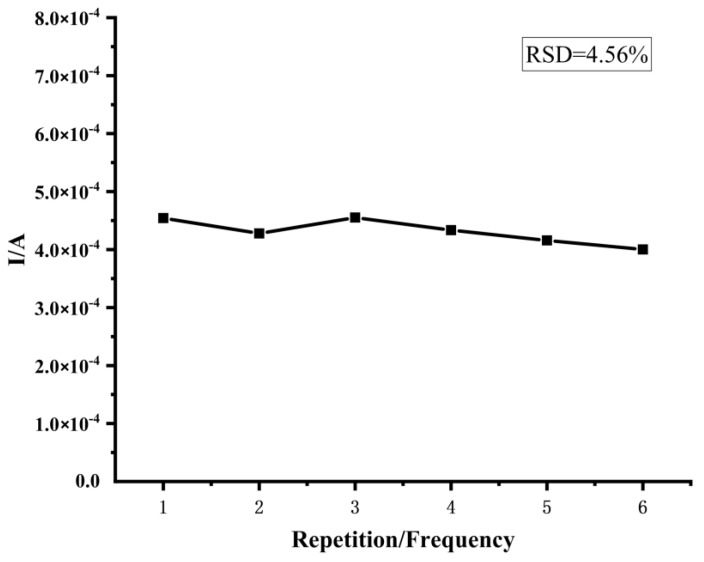
Results of the sensor repeatability test.

We found that the current response signal of the sensor did not show significant attenuation during the test, and the relative standard deviation of the results of the first six tests was 4.56% (Figure 5), indicating that the results obtained by the prepared sensor in the six consecutive tests were more accurate. 

### 3.5. Anti-Interference Test Results

Atrazine and metolachlor were added to 1 μmol/L cyromazine solution to prepare cyromazine containing 5 μmol/L, 10 μmol/L, 20 μmol/L, 30 μmol/L atrazine and metolachlor amine solution, respectively, and each group of solutions was tested. The results show that adding different concentrations of interfering substances has little effect on the test results, and the difference between the relative suppression and the original solution is less than 5% (Table 1 and Table 2). The above results show that this molecularly imprinted sensor has good anti-interference performance.

### 3.6. Recovery Analysis of Actual Samples

The prepared sensors were used to detect the recovery of tomato and cowpea and water samples, and each sample was repeated three times for each concentration. The results are shown in Table 3 and Table 4. The recovery rates of tomato and cowpea were 90.14% to 101.67% and 90.64% to 101.10%. The spiked recoveries of the water samples ranged from 91.1% to 108%, 114% to 118%, and 92.5% to 97.4%, respectively, and the relative standard deviations were all less than 6%. This shows that the molecularly imprinted sensor can meet the requirements of rapid detection of cyromazine in tomato and cowpea and water samples [22].

## 4. Discussion

Pretreatment of samples with a large number of organic solvents leads to the presence of many interfering substances in the treated samples, which have an unavoidable effect on the determination [23,24]. In this study, we selected two pesticides structurally similar to mefenoxam for interference experiments and found that the peak currents of the sensors did not change significantly after the addition of the interfering substances to the solution compared to those without the addition of the interfering substances. This suggests that molecularly imprinted polymers have the property of specific recognition. Li et al. [25] prepared a biosensor with a dual recognition system to detect lincomycin in meat products and the environment, with a detection limit of 1.6 × 10^−13^ mol/L, which meets the requirement of lincomycin in daily meat products. Dinali et al. synthesized a mesoporous molecularly imprinted polymer (core@mMIP) on the surface of silica nanoparticles to use it as a filler sorbent for microextraction for the selective determination of pesticides in apple juice. The sensor has been successfully applied to real samples of processed and fresh apple juice [26]. Li et al. successfully synthesized novel core–shell structured zeolite imidazole skeleton-8@ molecularly imprinted polymers by a surface imprinting technique and used them as sorbents for solid-phase extraction of organophosphorus pesticides. Under optimal conditions, the detection range of the method was from 1 to 200 µg/L. The recoveries of three different concentrations spiked in agricultural products (cauliflower, radish, pear, melon cauliflower, radish, pear, and muskmelon) ranged from 82.5% to 123.0% with the relative standard deviations below 8.24% [27]. These show that molecularly imprinted polymers have excellent recognition ability, and based on this property, molecularly imprinted sensors will be more rapidly developed and applied in the field of rapid detection of pesticide residues in food [28,29,30].

At present, the traditional pesticide residue detection technology has formed a complete set of detection systems and is widely used to detect pesticide residues in food [31,32,33]. Yu et al. [33] used a fluorescent quantitative method to detect paclobutrazol pesticide residues in apples. At a wavelength of 341 nm, the average recovery rate of the samples was 99.62%, and the relative standard deviation was 0.52%. Tsochatzis et al. [34] used matrix solid-phase dispersive extraction and high-performance liquid chromatography to detect eight pesticides commonly used in rice. The detection limits ranged from 0.002 mg/L to 0.2 mg/L with a relative standard deviation of less than 12%, which met the conditions for the detection of pesticide residues in rice. Although these traditional laboratory assays are characterized by high sensitivity and accurate results, they require complex sample pretreatment, have a high cost of detection, and are inconvenient in terms of portability, which hinder their further development. In this study, we prepared molecularly imprinted sensors with spiked recoveries ranging from 90% to 102% with relative deviations (RSDs) <5% using cyromazine as the template molecule and α-methacrylic acid as the functional monomer. The detection limit of the sensor was 0.083 mg/L, meeting the demand for the detection of cyromazine residues in fruits and vegetables. When we tested the repeatability of the sensor, we found that there was no significant difference (RSD < 5%) between the results obtained when the same sensor was used six times for the same concentration of the solution. This result indicates that the same sensor can be used at least six times, thus not only reducing the cost of pesticide residue testing but also reducing the number of contaminants generated during the testing process. The experimental results show that compared with traditional detection methods, the molecularly imprinted electrochemical sensor not only has accurate results and high sensitivity, but also reduces detection costs, is portable, suitable for large-scale applications, and has broad market prospects [35,36].

In this study, the sensor was only used to detect cyromazine residues in cowpea, tomato, and water samples, so the detection range needs to be expanded. Since cyromazine is also commonly used in the production and cultivation of edible agricultural products such as celery (*Apium graveolens* L.), cucumber (*Cucumis sativus* L.), and mango (*Mangifera indica* L.), this sensor can also be used to detect cyromazine residues in other foods to ensure food quality and consumer safety.

## Figures and Tables

**Table 1:** Test results of the sensor’s resistance to atrazine interference.

Samples	1 μmol/L Cyromazine Solution	1 μmol/L Cyromazine Solution + 5 μmol/L Atrazine	1 μmol/L Cyromazine Solution + 10 μmol/L Atrazine	1 μmol/L Cyromazine Solution + 20 μmol/L Atrazine	1 μmol/L Cyromazine Solution + 30 μmol/L Atrazine
Relative suppression	11.7%	9.07%	11.73%	8.64%	13.57%
Difference between relative suppression and stock solution	0.00%	2.63%	0.03%	3.06%	1.87%

**Table 2:** The test results of the sensor’s resistance to metolachlor interference.

Samples	1 μmol/ Cyromazine Solution	1 μmol/L Cyromazine Solution + 5 μmol/L Metolachlor	1 μmol/L Cyromazine Solution + 10 μmol/L Metolachlor	1 μmol/L Cyromazine Solution + 20 μmol/L Metolachlor	1 μmol/L Cyromazine Solution + 30 μmol/L Metolachlor
Relative suppression	17.40%	17.20%	19.07%	15.36%	16.27%
Difference between relative suppression and stock solution	0%	0.20%	1.67%	2.04%	0.93%

**Table 3:** Results of actual sample recovery.

Samples	Added (μmol/L)	Found (μmol/L)	Recovery (*n* = 3)	RSD (*n* = 3)
Tomato (*Lycopersicon esculentum*)	1	1	99.89%	4.16%
	2	2.03	101.67%	1.56%
	3	2.70	90.14	2.61%
Cowpea (*Vigna unguiculata*)	1	0.90	94.7%	4.98%
	2	2.02	101.10%	1.66%
	3	2.72	90.64%	2.52%

**Table 4:** Results of recovery of different water samples.

Samples	Added (μmol/L)	Found (μmol/L)	Recovery (*n* = 3)	RSD (*n* = 3)
River water	1	1.08	108%	2.39%
	2	1.82	91.1%	1.28%
	3	2.95	98.3%	3.39%
Water in the paddy field	1	1.16	116%	0.9%
	2	2.37	118%	2.33%
	3	3.43	114%	2.02%
Water in the botanical garden	1	0.925	92.5%	0.87%
	2	1.91	95.6%	5.79%
	3	2.92	97.4%	5.77%

## Data Availability

The datasets generated analyzed during the current study are available from the corresponding author on reasonable request.
